# DrugTar improves druggability prediction by integrating large language models and gene ontologies

**DOI:** 10.1093/bioinformatics/btaf360

**Published:** 2025-06-24

**Authors:** Niloofar Borhani, Iman Izadi, Ali Motahharynia, Mahsa Sheikholeslami, Yousof Gheisari

**Affiliations:** Department of Electrical and Computer Engineering, Isfahan University of Technology, Isfahan 84156-83111, Iran; Regenerative Medicine Research Center, Isfahan University of Medical Sciences, Isfahan 81746-73461, Iran; Department of Electrical and Computer Engineering, Isfahan University of Technology, Isfahan 84156-83111, Iran; Regenerative Medicine Research Center, Isfahan University of Medical Sciences, Isfahan 81746-73461, Iran; Isfahan Neuroscience Research Center, Isfahan University of Medical Sciences, Isfahan 81839-83434, Iran; Regenerative Medicine Research Center, Isfahan University of Medical Sciences, Isfahan 81746-73461, Iran; Department of Medicinal Chemistry, Isfahan University of Medical Sciences, Isfahan 81746-73461, Iran; Regenerative Medicine Research Center, Isfahan University of Medical Sciences, Isfahan 81746-73461, Iran; Department of Genetics and Molecular Biology, Isfahan University of Medical Sciences, Isfahan 81746-73461, Iran

## Abstract

**Motivation:**

Target discovery is crucial in drug development, especially for complex chronic diseases. Recent advances in high-throughput technologies and the explosion of biomedical data have highlighted the potential of computational druggability prediction methods. However, most current methods rely on sequence-based features with machine learning, which often face challenges related to hand-crafted features, reproducibility, and accessibility. Moreover, the potential of raw sequence and protein structure has not been fully investigated.

**Results:**

Here, we leveraged both protein sequence and structure using deep learning techniques, revealing that protein sequence, especially pre-trained embeddings, is more informative than protein structure. Next, we developed DrugTar, a high-performance deep learning algorithm integrating sequence embeddings from the ESM-2 pre-trained protein language model with gene ontologies to predict druggability. DrugTar achieved areas under the curve and precision–recall curve values of 0.94, outperforming state-of-the-art methods. In conclusion, DrugTar streamlines target discovery as a bottleneck in developing novel therapeutics.

**Availability and implementation:**

DrugTar is available as a web server at www.DrugTar.com. The data and source code are at https://github.com/NBorhani/DrugTar.

## 1 Introduction

Drug development for complex chronic diseases remains a challenge in current medicine. It is a costly, time-consuming process with a high failure rate, often due to the selection of incorrect targets for therapeutic intervention (Zhao *et al.* 2022). Hence, the primary focus in drug development lies in target discovery and validation. Despite the development of numerous FDA-approved drugs, these drugs target a minority of human proteins. Although a single protein can be targeted by multiple drugs, nearly 90% of proteins remain untargeted (Wishart *et al.* 2018, Abedi *et al.* 2021). This indicates that certain intrinsic characteristics make some proteins more suitable as drug targets (Raies *et al.* 2022). Predicting such a “druggability” score paves the way for identifying novel therapeutic targets. However, this task cannot be easily accomplished using a small set of protein features and relies on sophisticated methods that consider the combination of diverse parameters (Abedi *et al.* 2021). The development of these computational algorithms for druggability prediction provides an alternative strategy to the traditional time- and resource-consuming experimental approach.

While numerous computational methods have been developed for drug repositioning (Zhao *et al.* 2021, Borhani *et al.* 2022), few studies have focused on predicting novel drug targets. In our previous study (Abedi *et al.* 2021), we developed a machine learning method that utilized biochemical characteristics and network topology parameters to classify drug targets (DT) and non-drug targets (non-DT). Our findings indicated that biochemical characteristics are more informative for predicting druggability than network topology features. Most druggability prediction methods have been developed using machine learning techniques that rely on sequence-based features, like amino acid composition (AAC), as well as physicochemical properties such as hydrophobicity, polarity, and charge of amino acids extracted from sequences (Huang *et al.* 2010, Kumari *et al.* 2015, Jamali *et al.* 2016, Sikander *et al.* 2022). Ensemble learning methods, which combine multiple models using different machine learning techniques or protein features, have also been applied to improve druggability prediction (Lin *et al.* 2019, Charoenkwan *et al.* 2022, Raies *et al.* 2022). For instance, the SPIDER method (Charoenkwan *et al.* 2022) uses diverse sequence-based descriptors and well-known machine learning algorithms through a stacked ensemble learning approach.

The advancement of natural language processing (NLP) and large language models (LLMs) has enabled the learning of word embeddings, enhancing the understanding of biological sequence functions. However, applying NLP to protein sequences presents challenges, such as the lack of clear words and a wide range of sequence lengths (Ofer *et al.* 2021). To address these, Sun *et al.* (2018) used strategies such as single and 3-gram word2vec embeddings with different machine learning algorithms. Yu *et al.* (2022) were the first to apply deep learning techniques, including convolutional neural networks (CNN) and recurrent neural networks (RNN), to predict druggability from protein sequences. Additionally, QuoteTarget method (Chen *et al.* 2023) leveraged graph neural networks (GNN) and the pre-trained protein language model ESM1b to model protein sequences and predicted structures as graphs to enhance performance.

Despite the value of existing methods for predicting protein druggability, several significant barriers have limited their widespread adoption. These algorithms typically use sequence-based features, which do not fully leverage the comprehensive information within sequences or rely on manual biological feature extraction, making the process labor-intensive and time-consuming. On the other hand, recent deep learning methods have rarely surpassed the performance of machine learning strategies for druggability prediction, potentially due to the small size of the datasets. Furthermore, the introduced techniques are often unavailable as online tools, limiting their usability in experimental pipelines and hindering the assessment of their performance by independent investigators. Given these challenges and the critical gap in drug development, there is a clear need for a more robust, high-performance, and accessible method for predicting the tendency of proteins to be targeted by future therapeutic molecules. In this study, using the advantage of integrating pre-trained protein sequence embedding and protein ontologies, we developed DrugTar, a deep learning algorithm that demonstrates impressive functionality, superior performance compared with state-of-the-art methods, and consistent robustness across various assessments. This method, available as an online tool, facilitates target discovery, particularly in drug development for complex disorders.

## 2 Materials and methods

### 2.1 Dataset preparation

In this study, three datasets were created to assess various models: the “ProTar-I” dataset, which includes PDB files representing protein structures; the “ProTar-II” dataset, consisting of FASTA files containing protein sequences; and the “ProTar-II-Ind” dataset, an independent set of FASTA files not included in ProTar-II, used to evaluate the generalization of the models trained with ProTar-II (Supplementary Table S1, available as supplementary data at *Bioinformatics* online). The details of the datasets are described in the Supplementary Methods and Supplementary Tables S2–S4, available as supplementary data at *Bioinformatics* online.

### 2.2 DrugTar structure

We developed a deep learning-based method called DrugTar, which integrated Gene ontology (GO) terms and ESM-2 embedding vectors through a deep neural network (Supplementary Fig. S1, available as supplementary data at *Bioinformatics* online). DrugTar utilizes esm2_t33_650M_UR50D from ESM-2 (Lin *et al.* 2023), which was trained on nearly 650 million sequences, to extract amino acid representations. Protein sequences are fed into the pre-trained ESM-2 model, and the output from the 33rd layer, consisting of 1280 units, is extracted as the amino acid representation. For a protein of length *L*, the resulting sequence representation is a matrix $F\in \;R^{L\times 1280}$. Due to the high memory requirements for long protein sequences, tokens exceeding 1024 are removed. To compute the overall ESM-2 protein representation, the average of the amino acid embeddings is calculated as follows:


(1) $$F_{pr}=\frac{1}{L}\sum_{i=1}^{L}F(i,:)$$

where F(i,:) represents the *i*th amino acid representation, or the *i*th row of matrix F, and $F_{pr}\in R^{1\times 1280}$ is the resulting ESM-2 protein representation. To incorporate gene ontologies for druggability prediction, all three GO sub-ontologies, i.e. molecular function, cellular component, and biological process, are encoded into a binary vector, denoted as $GO_{\mathrm{pr}}\in B^{1\times 12610}$, and defined as follows:


(2) $${\mathrm{GO}}_{\mathrm{pr}}(1,\;i)=\{\begin{array}{c} \begin{array}{cc} 1\; & if\;\mathrm{protein}\;\mathrm{belongs}\;\mathrm{to}\;\mathrm{the}\;{i\mathrm{th}}_{}\;\mathrm{GO}\;\mathrm{term} \end{array}\\ \begin{array}{cc} 0\; & \mathrm{otherwise}\; \end{array} \end{array}$$

where $GO_{\mathrm{pr}}$ is a vector encoding gene ontologies. After obtaining both the ESM-2 protein representation and the GO binary vector, the protein representation is generated by concatenating them:


(3) $$R_{pr}=\mathrm{CONCAT}(F_{pr},{\mathrm{GO}}_{\mathrm{pr}})$$

where $R_{pr}$ is the protein representation used in DrugTar. Performing feature selection is essential in biological systems characterized by high-dimensional features to enhance classification and prevent overfitting (Kumari *et al.* 2015). To address this while ensuring an unbiased performance evaluation, the feature selection process was integrated with the classifier. In cross-validation (CV), feature selection was conducted exclusively on the training folds, ensuring the test fold remained unseen. The selected features were then used to train the model, which was subsequently evaluated on the test fold. Here, the SVM feature selection algorithm is applied, reducing $R_{pr}$ dimension to 1×4000. This reduced representation is passed through a deep neural network consisting of three hidden layers. Finally, a sigmoid activation function is applied to the output neuron to predict a continuous druggability score for the protein, ranging from zero to one.

### 2.3 DrugTar training and hyperparameter tuning

The DrugTar method, implemented in TensorFlow/Keras, was trained using the Adam optimizer with a binary cross-entropy loss function. A custom learning rate scheduler began at 0.0002, halving every 5 epochs to a minimum of 0.000025 after 15 epochs. The batch sizes were set to 32 for all datasets. The DNN architecture consisted of three hidden layers with 128, 64, and 32 units, respectively, using ReLU activation functions. A sigmoid function was applied in the final layer to predict a continuous druggability score between zero and one. To avoid overfitting, dropout with a drop probability of 0.5 was applied after the first and second dense layers, and L2 normalization with a penalty coefficient of 0.01 was used on the model weights. Furthermore, early stopping with a patience of five epochs was used. Batch normalization was applied after each dense layer.

### 2.4 Structure- and sequence-based models

The details of structure- and sequence-based models are described in the Supplementary Methods and Supplementary Figs S2–S6, available as supplementary data at *Bioinformatics* online.

### 2.5 Model assessment and implementation

To assess the efficacy of models' overall performance, a 10-fold CV protocol was used. The ProTar-I and ProTar-II datasets were validated through this 10-fold CV process. For each fold, the performance of the models was assessed using various metrics relevant to binary classification: area under the ROC curve (AUC), area under the precision-recall curve (AUPRC), precision, recall, accuracy, specificity, negative predictive value (NPV), F1, MCC, Kappa, diagnosis odds ratio (DOR), discriminant power (DP), and DKL.

Additionally, to evaluate model generalization, the ProTar-II-Ind dataset was used. The models were trained on the ProTar-II dataset and subsequently assessed using the independent test samples from the ProTar-II-Ind dataset. This approach was implemented to evaluate the generalization of the models, ensuring they perform well on unseen data.

Training and validation of DrugTar were implemented on a system equipped with an Intel Core i7-13620H CPU, an NVIDIA GeForce RTX 4060 GPU (8 GB), and 16 GB of RAM. The bidirectional encoder representations from transformers (BERT) and ESM-2 feature extraction and structure-based methods were implemented on a system with an Intel Core i7-4790K processor and 64 GB of RAM.

## 3 Results

### 3.1 Protein sequence is more informative than its 3D structure for druggability prediction

Both sequence and structural information can be exploited for druggability prediction. Protein functions are directly dictated by their 3D structures (Gao *et al.* 2020). Specifically, drugs bind to the 3D structure of their target proteins. Although protein sequence determines almost all protein characteristics, including the 3D structure, predicting structure from sequence requires sophisticated techniques. Therefore, we initially assumed it would be more straightforward to directly utilize structural features for druggability prediction rather than sequence data. Due to the lack of suitable datasets, the ProTar-I dataset was constructed, comprising refined structural information for 2248 proteins. This dataset includes both the atomic coordinates of 3D structures and the sequence information. For each protein, the highest resolution protein data bank (PDB) file with the longest sequence length was obtained from the RCSB PDB database (Berman *et al.* 2000), ensuring that the PDB file covers at least half of the full protein sequence. Proteins were isolated as monomers by excluding additional chains, water molecules, ions, ligands, and other heteroatoms. ProTar-I consists of PDB files for 1124 DTs and 1124 non-DTs. The DTs are FDA-approved drug targets, while the non-DTs are not targeted by any FDA-approved or experimental drugs. The specifications of ProTar-I are provided in Supplementary Table S1, available as supplementary data at *Bioinformatics* online.

To convert protein geometric 3D structures into a suitable format for deep learning methods, PDB files can be processed in several ways. One effective method is using point clouds, which reduce data volume compared to voxel grids. A point cloud consists of spatial data points extracted from PDB files, representing the 3D coordinates of atoms or amino acids. Here, amino acids were considered as points, defined by their alpha carbon coordinates. Additionally, amino acid coordinates were centralized to standardize protein positions. PointNet, a deep neural network designed to process 3D point clouds directly (Qi *et al.* 2017), was utilized with the constructed protein point clouds to forecast druggability, referred to as “PointNet-PC.” Since PointNet requires a fixed number of points, 400 amino acids were randomly selected for proteins with more than 400 amino acids, capturing the overall geometric shape of the protein. The number 400 was chosen because it is close to the average length of the ProTar-I samples. Furthermore, zero padding was used for proteins with fewer than 400 amino acids. To increase the training dataset 3-fold and ensure the model is invariant to rotation, data augmentation was conducted by applying random rotations during training.

**Figure 1.**
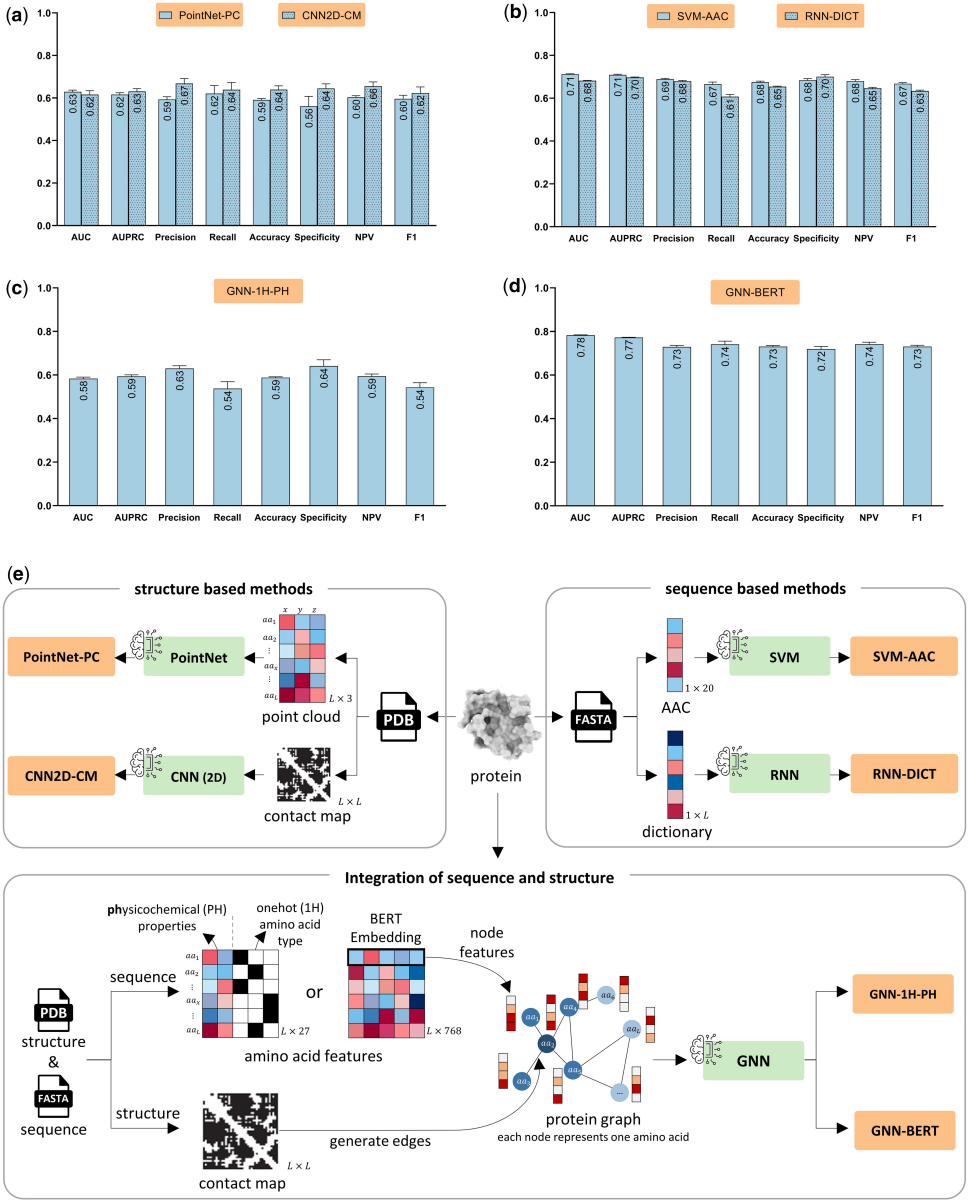
Comparison of sequence- and structure-based methods in druggability prediction. Both sequence- and structure-based approaches demonstrated moderate performance (a, b). The sequence and structure information were integrated into GNN models (c). Applying pre-trained BERT sequence embeddings as amino acid features led to improved performance in GNN-BERT compared to other methods, highlighting the importance of sequence information (d). Schematics of implemented methods are illustrated in panel (e). Data are presented as the mean ± standard deviation from five independent runs, each using a 10-fold cross-validation procedure.

In addition to point clouds, a protein structure can be represented by the distance map, a symmetric matrix illustrating distances between residues. By applying a distance threshold, this matrix is transformed into a binary protein contact map. This 2D representation effectively encodes the protein structure (Duarte *et al.* 2010). In this study, a threshold of 7 Å was used to obtain a contact map, and a 2D CNN was applied to predict druggability, referred to as “CNN2D-CM.” PointNet-PC and CNN2D-CM methods were evaluated using a 10-fold cross-validation (CV), and their performances were assessed using various performance indices. The PointNet-PC and CNN2D-CM methods moderately discriminated between DTs and non-DTs, showing comparable performance (Fig. 1a). Given that structure-based methods did not yield satisfactory results, we turned to sequence-based methods.

A variety of sequence features have been applied in druggability prediction (Charoenkwan *et al.* 2022, Sikander *et al.* 2022, Yu *et al.* 2022), with one of the most common being amino acid compositions, referred to as AAC. We began with AAC as a basic sequence feature and used a support vector machine (SVM) as a simple classifier. This method is referred to as “SVM-AAC.” Although AAC is a common sequence feature, it is limited by its lack of amino acid order. To address this limitation, “RNN-DICT” was developed. This method incorporates sequence information through dictionary encoding and uses bidirectional long short-term memory (Bi-LSTM) with attention mechanisms. These sequence-based methods demonstrated moderate performance in druggability prediction (Fig. 1b). Despite its simplicity, AAC provided valuable predictive information. Notably, these simple sequence-based algorithms slightly outperformed structure-based algorithms. It was observed that both sequence and structural information were moderately effective in discriminating between DTs and non-DTs.

To enhance performance, the next step was to integrate both sequence and structural data simultaneously. To this aim, a graph isomorphism network (GIN), a type of GNN known for its effectiveness in graph representation learning, was developed. For the first time, molecular graphs of proteins were constructed using real PDB files, with amino acids represented as nodes. The GIN propagates amino acid-level features across connected nodes based on the contact map matrix, generating protein-level feature representations for classification. Initially, one-hot encoding was used to represent amino acid types, along with seven physicochemical properties as outlined earlier (Meiler *et al.* 2001), specified as amino acid level features. The performance of this model, referred to as “GNN-1H-PH,” was not satisfactory (Fig. 1c). Recent studies have shown that features obtained from pre-trained language models can significantly enhance classification performance (Detlefsen *et al.* 2022). Therefore, BERT, a pre-trained language model specifically designed for protein sequences (Devlin *et al.* 2019, Rao *et al.* 2019) was utilized to obtain context-aware residue-level embedding vectors as node features. This method, referred to as “GNN-BERT,” leverages the power of pre-trained embeddings. While GNN-1H-PH was insufficiently discriminative, GNN-BERT demonstrated significantly superior performance compared to the mentioned methods across various indices, with a T-test *P*-value < 0.05 (Fig. 1d and e).

A Comparison between GNN-1H-PH and GNN-BERT models indicates that node features representing sequence information significantly influence GNN performance. To assess the contribution of structural information, the GNN-BERT architecture and hyperparameters were kept consistent while retraining and assessing the model by replacing the contact map with a random undirected graph having an equivalent number of edges as the original, along with an identity matrix. Interestingly, the model performance did not decline when using the random graph or identity contact matrix compared to the original graph (Table 1). This finding suggests that structural information does not significantly contribute to the classification power of GNN-BERT, whereas the BERT sequence embedding features play a crucial role. Taken together, these results demonstrate that protein sequence is more informative than structure for druggability prediction.

**Table 1.** Outcomes for different types of structure by different contact maps in the GNN-BERT.

Contact map	AUC	AUPRC	Precision	Recall	Accuracy	Specificity	NPV	F1
Original matrix	0.78	0.77	0.73	0.74	0.73	0.72	0.74	0.73
Random matrix	0.78	0.78	0.73	0.74	0.73	0.73	0.74	0.73
Identity matrix	0.78	0.78	0.72	0.75	0.73	0.70	0.74	0.73

### 3.2 Integrating pre-trained sequence embeddings with protein ontology data through deep learning enhances druggability prediction

**Figure 2.**
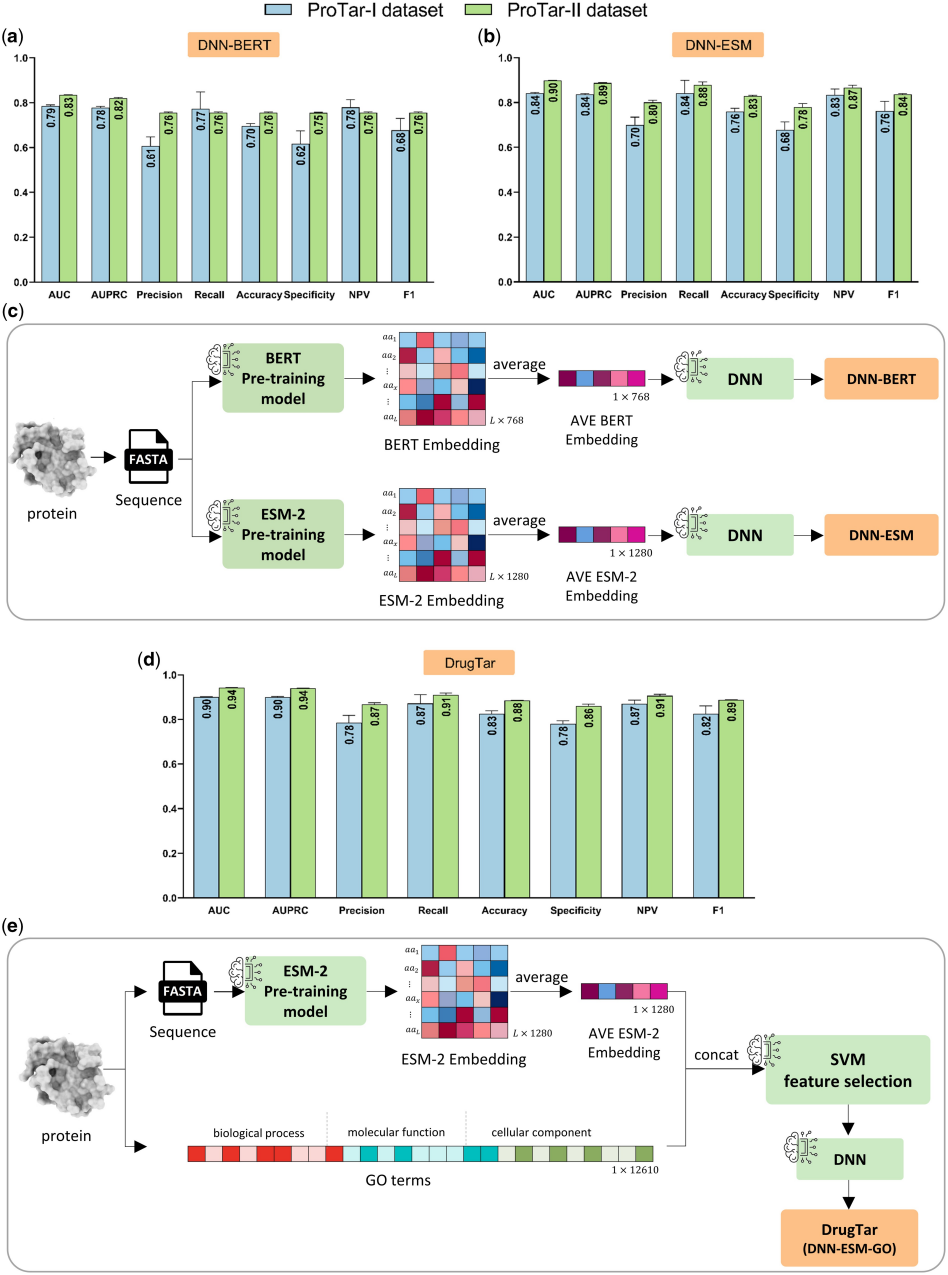
Enhancing druggability prediction with pre-trained sequence embeddings and gene ontologies. Using pre-trained sequence embeddings in a deep neural network yields satisfactory results. Despite only using sequence information, BERT-DNN achieved performance comparable to GNN-BERT, which requires both sequence and structure data, underscoring the importance of sequence. The ESM-2 model surpassed BERT in druggability prediction on ProTar-I and ProTar-II datasets (a, b). The performance of the proposed DrugTar model for druggability prediction based on integrating pre-trained sequence embedding and gene ontologies showed significant improvements across both datasets (d). Method schematics are shown in panels (c) and (e). Data are presented as the mean ± standard deviation from five independent runs, each using a 10-fold cross-validation procedure.

To further assess the role of protein structure in druggability prediction, a method named “DNN-BERT” was proposed to compare with GNN-BERT. DNN-BERT operates by averaging the residue-level embedding vectors generated by BERT and utilizing them in a deep neural network to classify DTs and non-DTs. Implementations on the ProTar-I dataset demonstrated that DNN-BERT, which relies solely on sequence information, performed comparably to GNN-BERT, which incorporates both sequence and structure data (Fig. 2a). This provides additional evidence that, within the methods implemented in this study, structural information does not offer further insights beyond sequence embeddings. The key advantage of DNN-BERT and similar models relying exclusively on sequence data is their broader applicability to proteins lacking structural data. This characteristic is valuable, as deep learning methods often yield better performance with larger datasets (LeCun *et al.* 2015). Hence, the ProTar-II dataset was constructed, containing sequence data for 2034 DTs and 2034 non-DTs without structural information (Supplementary Table S1, available as supplementary data at *Bioinformatics* online). DNN-BERT also showed strong performance on this dataset (Fig. 2a).

As indicated, the superiority of the DNN-BERT method lies in its use of the BERT language model, which has 110 million parameters and is pre-trained on approximately 31 million protein sequences (Devlin *et al.* 2019, Rao *et al.* 2019). To further improve predictions, we focused on utilizing a more advanced sequence pre-training model. ESM-2 offers enhanced sequence embeddings compared to BERT due to its larger number of parameters and a more extensive training dataset. The 33rd layer of ESM-2, esm2_t33_650M_UR50D, has been trained on approximately 65 million sequences (Lin *et al.* 2023). A new method, “DNN-ESM,” was constructed similarly to DNN-BERT, with ESM-2 replacing BERT. The 10-fold CV measurements showed that DNN-ESM outperformed DNN-BERT across all metrics (*P*-value < 0.05, Fig. 2b and c). Despite the performance improvement, ESM-2 has a limitation due to its high RAM requirements when processing long protein sequences. To address this, tokens exceeding 1024 in long protein were removed, as described in the previous study (Chen *et al.* 2023).

**Figure 3.**
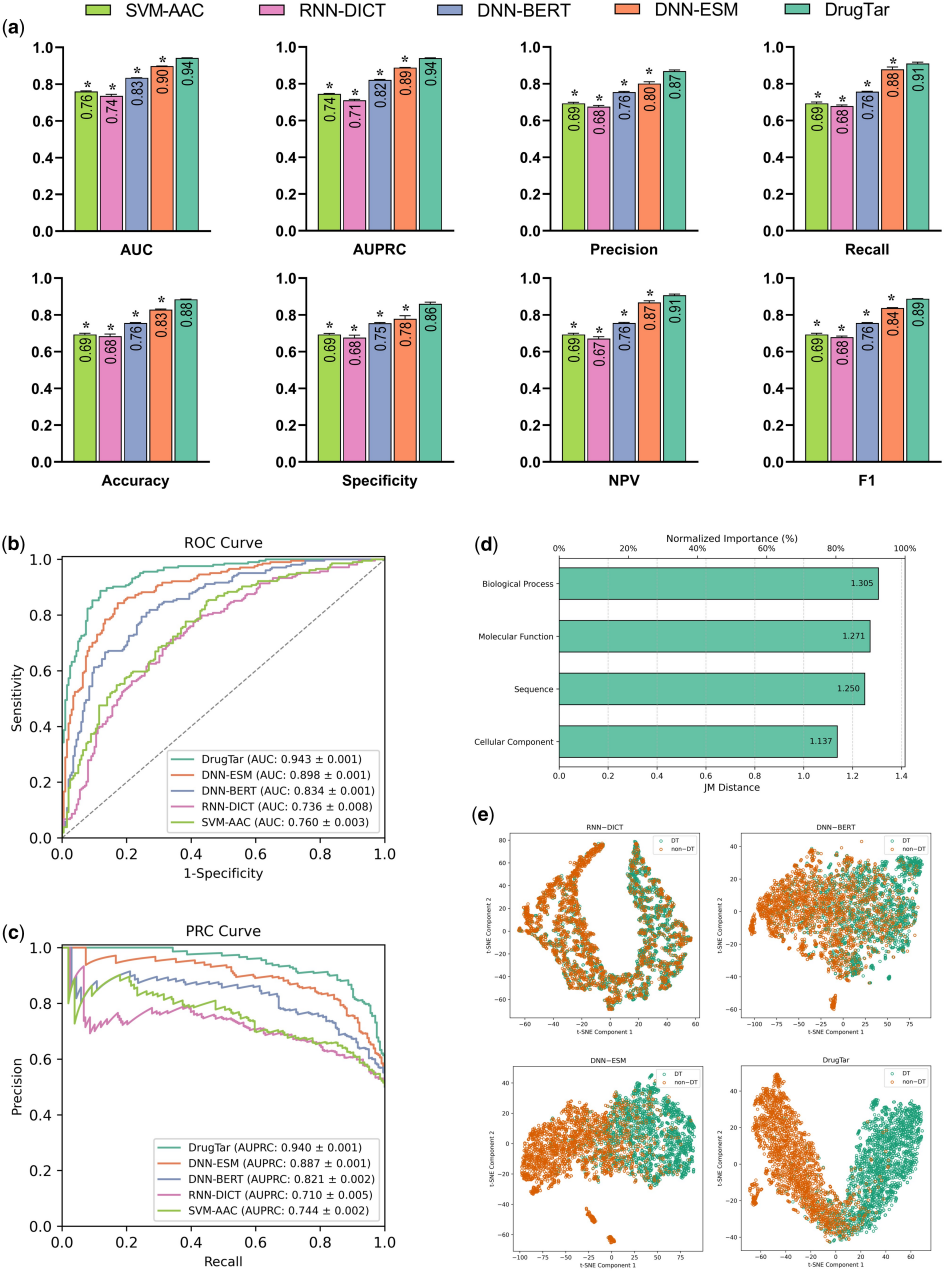
Performance evaluation of DrugTar and comparison with other methods on the ProTar-II dataset. The performance of the proposed DrugTar model was significantly superior to the examined sequence-based models (a). Performance is illustrated by the receiver operating characteristic (ROC) and precision–recall (PRC) curves, with each curve corresponding to a single run of 10-fold cross-validation (b, c). JM Distance analysis shows that sequence embeddings and GO terms effectively discriminate druggable and non-druggable proteins, highlighting the importance of GO terms integration in DrugTar (d). T-SNE visualization of protein latent vectors, where each marker represents a protein, showed that DrugTar provided superior discrimination between drug targets (DT) and non-drug targets (non-DT) proteins compared to other methods (e). Data are presented as the mean ± standard deviation from five independent runs.

Previous investigations have indicated that DTs often share common biological properties (Hu *et al.* 2019). GO terms describe different types of protein characteristics, including molecular function, cellular component, and biological process. Hence, we hypothesized that incorporating protein ontologies in the algorithm could enhance model performance. A new method, “DNN-ESM-GO,” was developed to integrate GO terms with ESM-2 embeddings. This method, renamed “DrugTar,” leverages the combined power of the ESM-2 embedding method and GO annotations within a deep neural network framework. In biological systems with high-dimensional features, effective feature selection is critical for improving classification accuracy and preventing overfitting (Kumari *et al.* 2015). Therefore, the SVM feature selection algorithm was used on the concatenation of ESM-2 embedding and GO terms in DrugTar. The feature selection process was integrated with the classifier to ensure unbiased performance evaluation (see Methods). Subsequently, a deep neural network with three hidden layers was utilized to predict protein druggability. DrugTar demonstrated robust performance on both the ProTar-I and ProTar-II datasets (Fig. 2d and e). The functionality of different algorithms was compared in the ProTar-II dataset, demonstrating the superiority of DrugTar with *P*-value < 0.05 for all indices (Fig. 3a–c). We assessed the discriminative power of feature categories, including sequence embeddings and three GO domains, using the Jeffries-Matusita (JM) distance (Fig. 3d). This analysis is critical, as features with low discriminative power limit the effectiveness of even the most robust classifiers. The results indicate that all feature types implemented in DrugTar are highly discriminative, regardless of the classifier. Notably, the GO terms were shown to be nearly as informative as the sequence embeddings, underscoring the value of integrating GO terms.

To further evaluate the discriminative power of the methods, T-SNE (T-distributed stochastic neighbor embedding) (Van der Maaten and Hinton 2008) was applied to the last dense layer before the output to visualize latent protein features in a 2D space. DrugTar provided better separation between DTs and non-DTs compared to other methods, highlighting its superior predictive capability (Fig. 3e).

### 3.3 DrugTar demonstrates robustness and stability, outperforming state-of-the-art methods

Various performance indices were calculated for the implemented methods, including those presented in earlier figures. Their definitions are provided in Supplementary Table S5, available as supplementary data at *Bioinformatics* online. Supplementary Table S6, available as supplementary data at *Bioinformatics* online presents the mean values of the performance indices along with their 95% confidence intervals (CIs) for all methods and datasets, following the Transparent Reporting of a Multivariable Prediction Model for Individual Prognosis or Diagnosis (TRIPOD) guidelines (Collins *et al.* 2015). The interpretation of the reference intervals for some indices has been previously detailed (Marateb *et al.* 2018). Accordingly, DrugTar demonstrated excellent balanced diagnosis accuracy (AUC = 0.94), excellent agreement with the gold standard [Cohen's kappa coefficient (Kappa) = 0.77], and a strong correlation with the gold standard [Matthews correlation coefficient (MCC) = 0.77] on the ProTar-II dataset. Furthermore, the Kullback–Leibler Divergence (DKL), a widely recognized metric for quantifying the difference between a target distribution and an estimated distribution, was calculated as 0.002, showing the model's reliability.

To assess the robustness of DrugTar, proteins in ProTar-II were randomly labeled as 1 or 0 using a binomial distribution, maintaining the same prevalence as in the dataset. This process was repeated five times to create multiple sets of randomly labeled proteins. As expected, DrugTar significantly outperformed the random classifier, demonstrating its reliable classification performance (Supplementary Fig. S7a, available as supplementary data at *Bioinformatics* online). The lift curve demonstrates that DrugTar not only achieves a significantly higher lift than the baseline, but also outperforms other sequence-based methods, demonstrating its superior ability to predict protein druggability (Supplementary Fig. S7b, available as supplementary data at *Bioinformatics* online).

**Figure 4.**
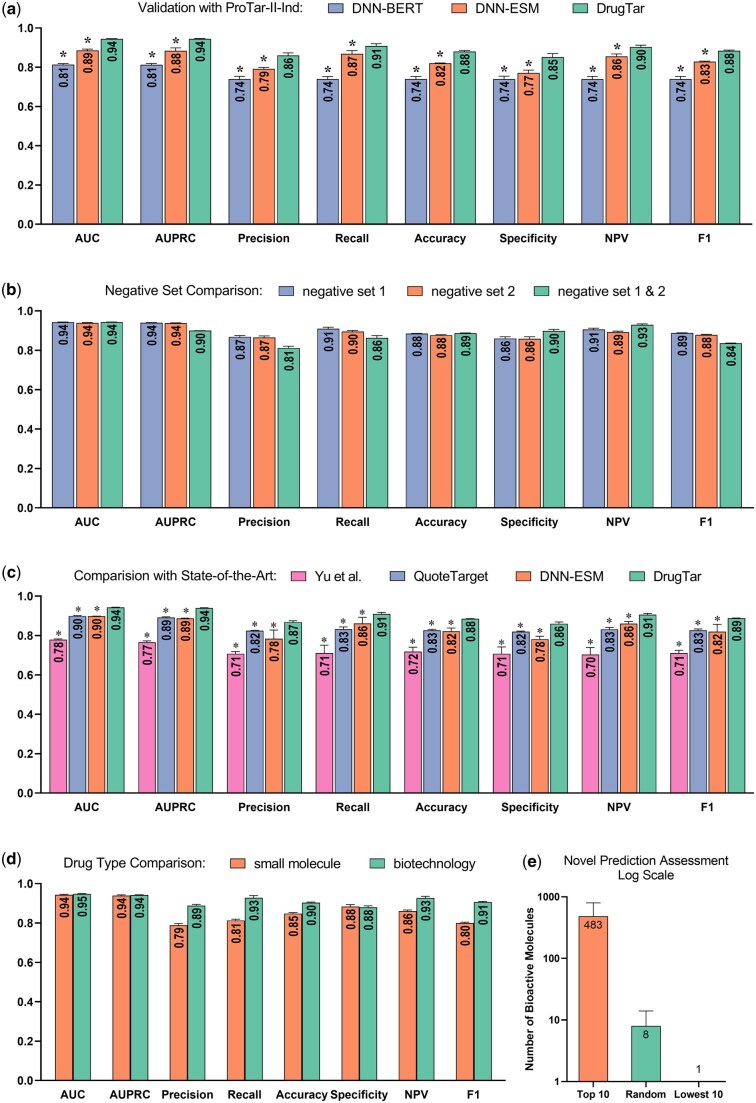
DrugTar demonstrated generalization and robustness in druggability prediction. DrugTar exhibited strong generalization and applicability on the independent test set ProTar-II-Ind (a). DrugTar showed stable and robust performance across various negative samples and imbalanced datasets (b). DrugTar consistently outperformed state-of-the-art methods on the ProTar-II dataset (c). DrugTar effectively predicts druggability scores for both biotechnology and small-molecule drug targets (d). The top 10 highest-scoring proteins, randomly selected sets of proteins, and the 10 lowest-scoring proteins predicted by DrugTar were validated using the ChEMBL database to assess the number of bioactive molecules, demonstrating the predictive strength of the model (e). Data are presented as the mean ± standard deviation from five independent runs (a, b, c, d) and as the mean ± mean squared error (MSE) on a logarithmic scale (e).

To further evaluate the functionality and generalization of DrugTar, an independent test set, ProTar-II-Ind, consisting of 225 DT and 225 non-DT samples, was created (Supplementary Table S1, available as supplementary data at *Bioinformatics* online). DrugTar was initially trained and optimized using the ProTar-II dataset, after which it was applied to predict druggability scores for unseen samples in the ProTar-II-Ind. These scores were comparable to those obtained in the 10-fold CV on the ProTar-II dataset, demonstrating the strong generalization of DrugTar (Fig. 4a). Additionally, DrugTar outperformed other methods developed in the current study across all indices on the ProTar-II-Ind (*P*-value < 0.05).

The Jamali dataset, established in 2016, contains 1224 DTs and 1319 non-DTs (Jamali *et al.* 2016). It is widely used in druggability prediction studies (Lin *et al.* 2019, Charoenkwan *et al.* 2022, Sikander *et al.* 2022, Yu *et al.* 2022). The DTs in this dataset are FDA-approved drug targets, while the non-DTs were extracted using methods outlined by Li and Lai (2007) and Bakheet and Doig (2009), where relevant family members of DTs were excluded from the non-DT list. To analyze the Jamali dataset, the average AAC of the Jamali and ProTar-II datasets was compared, revealing significantly higher AAC differences between DTs and non-DTs in the Jamali dataset (Supplementary Fig. S8, available as supplementary data at *Bioinformatics* online). This difference may be attributed to the method of selecting negative samples, suggesting that druggability prediction might be more straightforward in the Jamali dataset, potentially leading to overestimated performance. This underscores the significant impact of the method used for selecting negative samples on the evaluation.

To account for this, a second negative set of 2034 non-DTs, distinct from the negative set of ProTar-II, was extracted. DrugTar was evaluated using the same positive set but with this second negative set. The results showed comparable scores for both negative samples, indicating the robustness of DrugTar in handling negative samples. To further evaluate the robustness of DrugTar to an imbalanced dataset, both negative sets were combined, creating an imbalanced dataset (2034 DTs and 4068 non-DTs). Despite the imbalance, the performance of DrugTar remained strong (Fig. 4b).

Even with the bias in negative sample selection in the Jamali dataset, DrugTar was evaluated on this dataset due to its frequent use by other methods. DrugTar demonstrated excellent performance, outperforming both SPIDER (Charoenkwan *et al.* 2022) and Yu’s method (Yu *et al.* 2022) (Table 2). However, considering the significant impact of the dataset used for training and evaluation when comparing deep learning methods, we proceeded to compare state-of-the-art methods using the ProTar-II dataset to ensure a reliable and valuable comparison. Therefore, two current state-of-the-art methods were compared with DrugTar to verify that the features and network architecture used by DrugTar provide superior predictions of protein druggability. QuoteTarget (Chen *et al.* 2023) and the best-performing method from the Yu *et al.* (2022) study, both deep learning-based, were evaluated using 10-fold CV on the ProTar-II dataset. DrugTar significantly outperformed these methods across all indices (*P*-value < 0.05, Fig. 4c). Interestingly, the performance of DNN-ESM was comparable to that of QuoteTarget, which feeds ESM1b sequence embedding and contact maps predicted by ESM1b into a GNN.

**Table 2.** Performance comparison of DrugTar with state-of-the-art methods on the Jamali Dataset.^a^^,^^b^

Evaluation strategy	Method	AUC	Precision	Recall	Accuracy	F1
Cross-validation	DrugTar	**0.970**	**0.910**	**0.933**	**0.923**	**0.920**
	SPIDER	0.950	0.895	0.895	0.919	0.914
	Yu’s method	N.R.	0.905	0.890	0.900	0.896
Independent set	DrugTar	**0.959**	0.919	**0.908**	**0.916**	**0.913**
	SPIDER	0.902	0.857	0.857	0.989	0.899
	Yu’s method	N.R.	**0.936**	0.848	0.907	0.889

a Results were reported from the study of Yu’s method (Yu *et al.* 2022) and SPIDER (Charoenkwan *et al.* 2022).

^b^ Bold values represent the best (i.e. highest) value for each metric across methods. N.R. indicates that the value was not reported.

DrugTar was also tested separately on targets for biotechnology and small-molecule drugs. The performance was satisfactory for both groups (*P*-value < 0.05, Fig. 4d), demonstrating that DrugTar effectively predicts druggability scores for both groups of drug targets. These results confirm that DrugTar is a robust and valid method for predicting druggability.

To assess the novel DrugTar predictions, we trained DrugTar on the ProTar-II dataset to predict druggability scores for proteins excluded from both the training and testing phases, ensuring all test proteins were unseen during model training and validation. We extracted the top 10 highest-scoring proteins, five randomly selected sets of 10 proteins, and the 10 lowest-scoring proteins as predicted by DrugTar. Subsequently, we checked the ChEMBL database (Davies *et al.* 2015) to determine the number of bioactive molecules associated with these proteins. ChEMBL is a manually curated database of 2.4 million bioactive molecules with drug-like properties (Zdrazil *et al.* 2024). In this study, we consider the number of available bioactive molecules as an indicator of both the presence of an active site and the bioactivity of the target, with a higher count suggesting a greater likelihood of the target becoming a drug target. Among the top 10 highest-scoring proteins, 7 were found to be associated with several bioactive molecules. On average, these proteins interact with 482 ± 314 bioactive molecules. Notably, on average, only 1 out of 10 proteins in the five random sets interacts with 8 ± 6 bioactive molecules, and none of the 10 lowest-scoring proteins are associated with the bioactive molecules (Fig. 4e and Supplementary Tables S7–S9, available as supplementary data at *Bioinformatics* online). These findings provide strong evidence that DrugTar effectively differentiates between highly druggable and less druggable targets, aligning well with empirical data and demonstrating its utility in prioritizing proteins for drug discovery.

## 4 Discussion

Recent developments in high-throughput technologies have generated huge amounts of multi-omics data (Luo *et al.* 2021, Cui *et al.* 2022). This has enabled the identification of extensive lists of proteins involved in various diseases, leading to the discovery of numerous proteins associated with disease pathways (Macarron *et al.* 2011). However, a significant challenge limiting the clinical translation of these findings is the difficulty in identifying which disease-associated genes are appropriate targets for therapeutic intervention. This study is dedicated to addressing this critical issue, aiming to bridge the gap between disease-associated gene discovery and clinical application. To achieve this, an innovative high-performance method named DrugTar was developed to predict the most druggable targets among a list of genes involved in disease pathogenesis.

While some studies focus on protein pockets or use structural data for binding site prediction (Gainza *et al.* 2020, Tubiana *et al.* 2022), our approach filters a broad list of disease-related proteins to identify those with druggability potential. To the best of our knowledge, this study is the first to compare sequence and structure information in druggability prediction using various algorithms. Remarkably, the results demonstrated that sequence data are more informative than structural data. This finding aligns with recent studies showing that protein sequence, particularly sequence pre-trained embeddings, is more informative than protein structure for predicting protein function (Villegas-Morcillo *et al.* 2021, Tian *et al.* 2023) and protein interaction sites (Hosseini *et al.* 2024). While structure-based methods are valuable, sequence-based models offer significant advantages, including enhanced accessibility and simpler data processing. However, we acknowledge that the superiority of sequence versus structure for druggability prediction is limited to the methods used and cannot be generalized.

The performance of SVM-AAC, as a simple machine learning method, was comparable to, and in some cases even surpassed, sophisticated deep learning methods, including GNN-1H-PH and RNN-DICT. This is in line with the study of Yu *et al.* who found that their deep learning method for druggability prediction was not better than machine learning algorithms (Yu *et al.* 2022). This unexpected finding can be explained by the fact that machine learning often outperforms deep learning on smaller datasets due to their simplicity and lower risk of overfitting. Conversely, as dataset size increases, deep learning models tend to excel because they can learn complex patterns from large amounts of data (Ching *et al.* 2018). Labeled data is often scarce in biological settings, limiting the applicability of deep learning methods. Using pre-trained models on large, diverse datasets and then fine-tuning them on smaller, problem-specific datasets is a wise approach to address this problem (Alley *et al.* 2019, Detlefsen *et al.* 2022). In this study, using pre-trained models like BERT or ESM-2 for sequence embedding significantly improved the distinction between DTs and non-DTs, consistent with previous findings that pre-trained sequence embeddings enhance performance in other biological applications (Villegas-Morcillo *et al.* 2021, Jha *et al.* 2022). Importantly, these pre-trained models are trained using self-supervised tasks without access to task-specific labels, ensuring that improvements arise from deep and generalizable sequence information rather than label leakage. Thus, while the same sequences may be included in pre-training and fine-tuning phases, no druggability labels are available to the model during pre-training, preventing data leakage or bias.

To enhance druggability prediction, DrugTar integrates ESM-2 sequence embeddings and protein ontologies through a deep neural network. Due to the large number of GO terms used in this algorithm, an SVM-based feature selection method was used to avoid overfitting. Among the various available biological features, we selected protein ontologies as they are informative and represent intrinsic protein characteristics. Furthermore, they are disease-independent and can be easily and robustly accessed. Their hierarchical organization and well-annotation provide additional advantages. The improvements achieved by incorporating GO terms are consistent with the findings of Bakheet *et al.* (Bakheet and Doig 2009), who identified specific GO terms associated with the tendency of proteins to be targeted by drugs. In line, we previously found that biochemical features of proteins are informative for druggability prediction (Abedi *et al.* 2021). Moreover, the JM distance calculation showed that GO terms are nearly as important as the sequence in predicting druggability, improving the balanced diagnostic accuracy to excellent. However, if GO terms are unavailable, the DrugTar can still predict druggability using sequence data, ensuring its robustness and applicability.

The appropriate selection of informative features in DrugTar, such as GO terms, allowed us to use a simple classifier. Indeed, although we experimented with various sophisticated integration and classification algorithms, the simple 3-layer DNN implemented in DrugTar consistently delivered superior performance. This underscores the fact that simple classifiers with appropriate features can achieve excellent results (Asgari and Mofrad 2015). Additionally, the simplicity of the classifier significantly reduces the computation cost.

The availability of a representative labeled dataset is a bottleneck in developing deep learning algorithms, particularly for classification. Any bias in the dataset may result in over- or underestimating the performance of the classifier. We demonstrated that a widely used dataset in developing druggability prediction methods (Jamali *et al.* 2016) suffers from bias when selecting negative samples. Hence, the performance of the validated algorithms using this dataset remains to be further assessed with other datasets. In the present study, the functionality of DrugTar was assessed using different generated datasets, including ProTar-I, ProTar-II, and ProTar-II-Ind. DrugTar consistently demonstrated strong performance across all datasets and surpassed state-of-the-art methods. Furthermore, the consistent performance across different negative sets demonstrates the robustness of DrugTar in handling negative samples. Additionally, when the size of negative samples was twice that of positive samples, the performance of DrugTar remained high, indicating the robustness of this algorithm to unbalanced data. Additionally, DrugTar demonstrated reliable and discriminative performance for both small-molecule drugs and biotechnology pharmaceutical targets. Notably, the validity of the top-ranked predictions was confirmed through empirical data from ChEMBL. These assessments clearly indicate the robust high performance of DrugTar for druggability prediction.

In conclusion, we developed DrugTar, a deep learning-based method that integrates ESM-2 sequence embedding with protein ontologies, achieving an impressive AUC and AUPRC of 0.94. Extensive assessments across multiple datasets and comparisons with alternative tools demonstrated its robustness and effectiveness, reflected in excellent diagnostic accuracy, strong agreement, and high correlation with the gold standard. The novel prediction capabilities of this method suggest its applicability in drug target prediction, particularly for complex disorders. By streamlining the selection of candidate proteins for pre-clinical examinations, DrugTar efficiently translates biological findings into clinical applications, thereby facilitating the drug development process. To enhance accessibility, we developed an online tool for DrugTar, available at http://DrugTar.com.

## Supplementary Material

btaf360_Supplementary_Data

## Data Availability

The data and source code related to the training and evaluation of the DrugTar model are publicly available at https://github.com/NBorhani/DrugTar and have been archived at https://doi.org/10.5281/zenodo.15359346. DrugTar is also available as a web server at www.DrugTar.com. The web server enables users to predict druggability scores for proteins using the DrugTar method.

## References

[btaf360-B1] Abedi M , Marateb HR, Mohebian MR et al Systems biology and machine learning approaches identify drug targets in diabetic nephropathy. Sci Rep 2021;11:23452. 34873190 10.1038/s41598-021-02282-3 PMC8648918

[btaf360-B2] Alley EC , Khimulya G, Biswas S et al Unified rational protein engineering with sequence-based deep representation learning. Nat Methods 2019;16:1315–22. 31636460 10.1038/s41592-019-0598-1 PMC7067682

[btaf360-B3] Asgari E , Mofrad MRK. Continuous distributed representation of biological sequences for deep proteomics and genomics. PLoS One 2015;10:e0141287. 26555596 10.1371/journal.pone.0141287 PMC4640716

[btaf360-B4] Bakheet TM , Doig AJ. Properties and identification of human protein drug targets. Bioinformatics 2009;25:451–7. 19164304 10.1093/bioinformatics/btp002

[btaf360-B5] Berman HM , Westbrook J, Feng Z et al The Protein Data Bank. Nucleic Acids Res 2000;28:235–42. 10592235 10.1093/nar/28.1.235 PMC102472

[btaf360-B6] Borhani N , Ghaisari J, Abedi M et al A deep learning approach to predict inter-omics interactions in multi-layer networks. BMC Bioinformatics 2022;23:53. 35081903 10.1186/s12859-022-04569-2 PMC8793231

[btaf360-B7] Charoenkwan P , Schaduangrat N, Lio' P et al Computational prediction and interpretation of druggable proteins using a stacked ensemble-learning framework. iScience 2022;25:104883. 36046193 10.1016/j.isci.2022.104883 PMC9421381

[btaf360-B8] Chen J et al QuoteTarget: a sequence-based transformer protein language model to identify potentially druggable protein targets. Protein Sci 2023;32:1–14. 10.1002/pro.4555 PMC9878469 36564866

[btaf360-B9] Ching T , Himmelstein DS, Beaulieu-Jones BK et al Opportunities and obstacles for deep learning in biology and medicine. J R Soc Interface 2018;15:20170387. 29618526 10.1098/rsif.2017.0387 PMC5938574

[btaf360-B10] Collins GS , Reitsma JB, Altman DG et al Transparent reporting of a multivariable prediction model for individual prognosis or diagnosis (TRIPOD): the TRIPOD statement. BMC Med 2015;13:1. 25563062 10.1186/s12916-014-0241-z PMC4284921

[btaf360-B11] Cui M , Cheng C, Zhang L et al High-throughput proteomics: a methodological mini-review. Lab Invest 2022;102:1170–81. 35922478 10.1038/s41374-022-00830-7 PMC9362039

[btaf360-B12] Davies M , Nowotka M, Papadatos G et al ChEMBL web services: streamlining access to drug discovery data and utilities. Nucleic Acids Res 2015;43:W612–20. 25883136 10.1093/nar/gkv352 PMC4489243

[btaf360-B13] Detlefsen NS , Hauberg S, Boomsma W et al Learning meaningful representations of protein sequences. Nat Commun 2022;13:1914. 35395843 10.1038/s41467-022-29443-w PMC8993921

[btaf360-B14] Devlin J, Chang MW, Lee K et al BERT: pre-training of deep bidirectional transformers for language understanding. In: *Proceedings of the 2019 Conference of the North*. Stroudsburg, PA, USA: Association for Computational Linguistics, 2019, 4171–86.

[btaf360-B15] Duarte JM , Sathyapriya R, Stehr H et al Optimal contact definition for reconstruction of contact maps. BMC Bioinformatics 2010;11:283. 20507547 10.1186/1471-2105-11-283 PMC3583236

[btaf360-B16] Gainza P , Sverrisson F, Monti F et al Deciphering interaction fingerprints from protein molecular surfaces using geometric deep learning. Nat Methods 2020;17:184–92. 31819266 10.1038/s41592-019-0666-6

[btaf360-B17] Gao W , Mahajan SP, Sulam J et al Deep learning in protein structural modeling and design. Patterns (N Y) 2020;1:100142. 33336200 10.1016/j.patter.2020.100142 PMC7733882

[btaf360-B18] Hosseini S , Golding GB, Ilie L et al Seq-InSite: sequence supersedes structure for protein interaction site prediction. Bioinformatics 2024;40:1–11. 10.1093/bioinformatics/btad738 PMC10796176 38212995

[btaf360-B19] Hu Y , Zhao T, Zhang N et al A review of recent advances and research on drug target identification methods. Curr Drug Metab 2019;20:209–16. 30251599 10.2174/1389200219666180925091851

[btaf360-B20] Huang C , Zhang R, Chen Z et al Predict potential drug targets from the ion channel proteins based on SVM. J Theor Biol 2010;262:750–6. 19903486 10.1016/j.jtbi.2009.11.002

[btaf360-B21] Jamali AA , Ferdousi R, Razzaghi S et al DrugMiner: comparative analysis of machine learning algorithms for prediction of potential druggable proteins. Drug Discov Today 2016;21:718–24. 26821132 10.1016/j.drudis.2016.01.007

[btaf360-B22] Jha K , Saha S, Singh H et al Prediction of protein–protein interaction using graph neural networks. Sci Rep 2022;12:8360. 35589837 10.1038/s41598-022-12201-9 PMC9120162

[btaf360-B23] Kumari P , Nath A, Chaube R et al Identification of human drug targets using machine-learning algorithms. Comput Biol Med 2015;56:175–81. 25437231 10.1016/j.compbiomed.2014.11.008

[btaf360-B24] LeCun Y , Bengio Y, Hinton G et al Deep learning. Nature 2015;521:436–44. 26017442 10.1038/nature14539

[btaf360-B25] Li Q , Lai L. Prediction of potential drug targets based on simple sequence properties. BMC Bioinformatics 2007;8:353. 17883836 10.1186/1471-2105-8-353 PMC2082046

[btaf360-B26] Lin J , Chen H, Li S et al Accurate prediction of potential druggable proteins based on genetic algorithm and Bagging-SVM ensemble classifier. Artif Intell Med 2019;98:35–47. 31521251 10.1016/j.artmed.2019.07.005

[btaf360-B27] Lin Z , Akin H, Rao R et al Evolutionary-scale prediction of atomic-level protein structure with a language model. Science 2023;379:1123–30. 36927031 10.1126/science.ade2574

[btaf360-B28] Luo H , Li M, Yang M et al Biomedical data and computational models for drug repositioning: a comprehensive review. Brief Bioinform 2021;22:1604–19. 32043521 10.1093/bib/bbz176

[btaf360-B29] Macarron R , Banks MN, Bojanic D et al Impact of high-throughput screening in biomedical research. Nat Rev Drug Discov 2011;10:188–95. 21358738 10.1038/nrd3368

[btaf360-B30] Marateb HR , Mohebian MR, Javanmard SH et al Prediction of dyslipidemia using gene mutations, family history of diseases and anthropometric indicators in children and adolescents: the CASPIAN-III study. Comput Struct Biotechnol J 2018;16:121–30. 30026888 10.1016/j.csbj.2018.02.009 PMC6050175

[btaf360-B31] Meiler J , Zeidler A, SchmSchke F et al Generation and evaluation of dimension-reduced amino acid parameter representations by artificial neural networks. J. Mol. Model 2001;7:360–9.

[btaf360-B32] Ofer D , Brandes N, Linial M et al The language of proteins: NLP, machine learning & protein sequences. Comput Struct Biotechnol J 2021;19:1750–8. 33897979 10.1016/j.csbj.2021.03.022 PMC8050421

[btaf360-B33] Qi CR, Su H, Mo K et al PointNet: deep learning on point sets for 3D classification and segmentation. In: *2017 IEEE Conference on Computer Vision and Pattern Recognition (CVPR), Honolulu, HI, USA*. IEEE, 2017, 77–85.

[btaf360-B34] Raies A , Tulodziecka E, Stainer J et al DrugnomeAI is an ensemble machine-learning framework for predicting druggability of candidate drug targets. Commun Biol 2022;5:1291. 36434048 10.1038/s42003-022-04245-4 PMC9700683

[btaf360-B35] Rao R et al Evaluating protein transfer learning with TAPE. Adv Neural Inf Process Syst 2019;32:1–13. PMC7774645 33390682

[btaf360-B36] Sikander R , Ghulam A, Ali F et al XGB-DrugPred: computational prediction of druggable proteins using eXtreme gradient boosting and optimized features set. Sci Rep 2022;12:5505. 35365726 10.1038/s41598-022-09484-3 PMC8976041

[btaf360-B37] Sun T , Lai L, Pei J et al Analysis of protein features and machine learning algorithms for prediction of druggable proteins. Quant Biol 2018;6:334–43.

[btaf360-B38] Tian X, Wang Z, Yang KK et al Sequence vs. Structure: delving deep into data-driven protein function prediction. bioRxiv, 2023, 2023–04.

[btaf360-B39] Tubiana J , Schneidman-Duhovny D, Wolfson HJ et al ScanNet: an interpretable geometric deep learning model for structure-based protein binding site prediction. Nat Methods 2022;19:730–9. 35637310 10.1038/s41592-022-01490-7

[btaf360-B40] Van der Maaten L , Hinton G. Visualizing data using t-SNE laurens. J Mach Learn Res 2008;9:2579–2605.

[btaf360-B41] Villegas-Morcillo A , Makrodimitris S, van Ham RCHJ et al Unsupervised protein embeddings outperform hand-crafted sequence and structure features at predicting molecular function. Bioinformatics 2021;37:162–70. 32797179 10.1093/bioinformatics/btaa701 PMC8055213

[btaf360-B42] Wishart DS , Feunang YD, Guo AC et al DrugBank 5.0: a major update to the DrugBank database for 2018. Nucleic Acids Res 2018;46:D1074–82. 29126136 10.1093/nar/gkx1037 PMC5753335

[btaf360-B43] Yu L , Xue L, Liu F et al The applications of deep learning algorithms on in silico druggable proteins identification. J Adv Res 2022;41:219–31. 36328750 10.1016/j.jare.2022.01.009 PMC9637576

[btaf360-B44] Zdrazil B , Felix E, Hunter F et al The ChEMBL database in 2023: a drug discovery platform spanning multiple bioactivity data types and time periods. Nucleic Acids Res 2024;52:D1180–92. 37933841 10.1093/nar/gkad1004 PMC10767899

[btaf360-B45] Zhao K , Shi Y, So H-C et al Prediction of drug targets for specific diseases leveraging gene perturbation data: a machine learning approach. Pharmaceutics 2022;14:234. 35213968 10.3390/pharmaceutics14020234 PMC8878225

[btaf360-B46] Zhao T , Hu Y, Valsdottir LR et al Identifying drug–target interactions based on graph convolutional network and deep neural network. Brief Bioinform 2021;22:2141–50. 32367110 10.1093/bib/bbaa044

