# Supplementary material for: DrugTar improves druggability prediction by integrating large language models and gene ontologies
Source: Bioinformatics. 2025 Jun 24;41(7):btaf360. doi: 10.1093/bioinformatics/btaf360 (PMC12312791; doi:10.1093/bioinformatics/btaf360)
Supplement: btaf360_Supplementary_Data [file btaf360_supplementary_data.zip › btaf360_Supplementary_Data/Supplementary Methods & Figures.docx]

**Supplementary Methods & Figures**

**DrugTar Improves Druggability Prediction by Integrating Large Language Models and Gene Ontologies**

**Niloofar Borhani ^1,2^, Iman Izadi ^1^*, Ali Motahharynia ^2,3^, Mahsa Sheikholeslami ^2,4^, Yousof Gheisari ^2,5^***

1. Department of Electrical and Computer Engineering, Isfahan University of Technology, Isfahan, Iran
2. Regenerative Medicine Research Center, Isfahan University of Medical Sciences, Isfahan, Iran
3. Isfahan Neuroscience Research Center, Isfahan University of Medical Sciences, Isfahan, Iran
4. Department of Medicinal Chemistry, Isfahan University of Medical Science, Isfahan, Iran
5. Department of Genetics and Molecular Biology, Isfahan University of Medical Sciences, Isfahan, Iran

*** Corresponding Authors:**

Iman Izadi, Ph.D. Department of Electrical and Computer Engineering, Isfahan University of Technology, Isfahan, 84156-83111, Iran Tel: +98 313 391 9047. Email: iman.izadi@iut.ac.ir

Yousof Gheisari MD, PhD. Regenerative Medicine Research Center, Isfahan University of Medical Sciences, Isfahan, 81746-73461, Iran Tel/Fax: +98-3136687087. Email: [ygheisari@med.mui.ac.ir](mailto:ygheisari@med.mui.ac.ir)

# Supplementary Methods

## Dataset preparation

ProTar-I. To create the dataset capturing protein structure for drug target prediction algorithms, PDB files were obtained from the RCSB PDB database (Berman, 2000), ensuring that the PDB file covers at least half of the full protein sequence. When multiple PDB files existed for a protein, the file with the longest residue length and the best resolution was selected (Supplementary Table 7). Since PDB files often contain multiple chains, we isolated monomer proteins by removing other chains. Furthermore, water molecules, ions, ligands, and other heteroatoms were eliminated. This dataset comprises 1,224 PDBs for DT proteins and 1,224 PDBs for non-DT proteins. The DTs are FDA-approved drug targets, while the non-DTs are not targeted by any FDA-approved or experimental drugs, according to the DrugBank database (version 5.1, (Wishart, 2006; Knox *et al.*, 2024)).

ProTar-II and ProTar-II-Ind. The approved DT human proteins were downloaded from the DrugBank database (version 5.1, (Wishart, 2006; Knox *et al.*, 2024)). Proteins not associated with drugs were obtained by excluding targets, enzymes, carriers, and transporters related to any drugs listed in the DrugBank database from a complete list of all reviewed human proteins (n = 20433) sourced from the UniProt database (Bateman *et al.*, 2023). Out of 2,291 approved targets in DrugBank, 2,261 were found in the list of reviewed UniProt IDs, and their sequences were extracted. Then, proteins with sequence lengths smaller than 50 amino acids or greater than 10,000 amino acids were excluded. Employing negative sampling equal to 1 resulted in a final set comprising 2,259 DT proteins and 2,259 non-DT proteins. Protein sequences were extracted in FASTA format from the UniProt database. From this set, 90% of the data was used to construct ProTar-II, consisting of 2,034 DT proteins and 2,034 non-DT proteins (Supplementary Table 8). ProTar-II was split into 9:1 subsets for training and testing models using 10-fold cross-validation. The remaining 10%, consisting of 225 DTs and 225 non-DTs, was used to construct ProTar-II-Ind, a dataset for independent validation to assess model generalization (Supplementary Table 9). We set an 80% identity threshold for clustering the sequences using CD-HIT, ensuring no sequences with more than 20% identity overlap between the ProTar-II and ProTar-II-Ind. The GO annotation information, including biological process, molecular function, and cellular components were extracted from the GO database (gaf-version: 2.2, (Ashburner *et al.*, 2000; Aleksander *et al.*, 2023)). Annotations assigned to root terms, GO:0008150 (biological process), GO:0003674 (molecular function), and GO:0005575 (cellular component), were omitted to ensure the specificity.

## Structure- and sequence-based models

PointNet-PC. PointNet (Charles *et al.*, 2017) is an effective framework for processing raw point cloud data extracted from protein PDB files. Point order invariance and transformation invariance, are two essential properties of PointNet that make it highly compatible with the 3D structure of proteins. In this study, amino acids are considered as points, and their corresponding coordinates are the coordinates of the Cα atoms. We replicated the network architecture from the original paper (Charles *et al.*, 2017) and reduced the number of weights in each layer by half to better suit the druggability prediction and reduce overfitting (Supplementary Fig. 2).

CNN2D-CM. The CNN2D-CM model applies two-dimensional CNN on the protein residue contact map. To create contact maps, residues are considered to be in contact if the Euclidean distance between their corresponding Cα atoms is less than $7 Å$ (Gligorijević *et al.*, 2021; Huang *et al.*, 2020). The CNN architecture consists of two convolutional layers, with filter sizes of 5×5×32 and 5×5×64, both using ReLU activation functions (Supplementary Fig. 3).

SVM-AAC. In the SVM-AAC model, the AAC vector, representing the frequency of 20 amino acids in the protein sequence, is used as an input feature for the SVM classifier to predict druggability.

RNN-DICT. RNNs are suitable for tasks where the order of information is crucial, such as sequence classification. Long short-term memory (LSTM) networks are particularly effective at retaining important information from earlier parts of the sequence, improving contextual understanding and predictions. The RNN-DICT method employs dictionary-encoded sequences and utilizes bi-LSTMs with attention mechanisms. A key feature of this model is an attention layer, which utilizes all output states from the bi-LSTMs to enhance prediction accuracy (Supplementary Fig. 4).

Graph Isomorphism Network. The GIN (Xu *et al.*, 2019) is an effective method for processing protein graphs and integrating both sequence and structural information. In this study, the molecular graph of proteins is constructed using real PDB files. To this aim, amino acids are defined as nodes and there is an edge between amino acids if the distance between their $C_{\alpha}$ atoms is less than $7 Å$(Gligorijević *et al.*, 2021; Huang *et al.*, 2020). The GIN processes the adjacency matrix (contact map) along with amino acid features as input, learning a representation vector for both individual amino acids and the overall protein.

GIN iteratively updates the representation of each amino acid by aggregating the representations of its neighbors. Hence, the representation of the $i_{th}$ amino acid obtained by the $k_{th}$ layer of GIN is defined as follows:

| $a_{i}^{\left( k \right)}=AGGREGATE^{(k)}\left( e_{iv}h_{v}^{(k-1)} \vert\vert v\in N(i) \right)$ | (1) |
| --- | --- |
| $h_{i}^{\left( k \right)}=COMBINE^{(k)}\left( h_{i}^{(k-1)},a_{i}^{\left( k \right)} \right)$ | (2) |

where $N(i)$ denotes the neighbors of the $i_{th}$ amino acid, and $h_{i}^{\left( k \right)}$is the representation of the $i_{th}$ amino acid at the $k_{th}$ GIN layer. Notably, the implemented method includes two GIN layers ($k=0,1,2$), where $h_{i}^{0}$ refers to the initial amino acid feature. This feature can either be one-hot encoding with physicochemical properties in the GNN-1H-PH model, or BERT embeddings in the GNN-BERT model. The seven physicochemical properties of amino acids were outlined previously (Meiler *et al.*, 2001). We opted for BERT embeddings over ESM-2 embeddings due to hardware limitations, as embedding all amino acids, especially in long proteins, would require significant RAM resources. The $e_{iv}$ denotes the feature edge between the $i_{th}$ and $v_{th}$ amino acids, which are defined as:

| $e_{iv}=\frac{1}{{dist (i,v)}^{2}+\varepsilon}$ | (3) |
| --- | --- |

where $dist(i,v)$ denotes the distance between the $C_{\alpha}$ atoms of the $i_{th}$ and $v_{th}$ residue, and $\varepsilon$ is set to ${10}^{-6}$. For the $AGGREGATE^{(k)}$ operation in Eq. 1, the sum operation is used, and for $COMBINE^{(k)}$ operation in Eq. 2, a linear function is applied, the same as in the previous study (Xu *et al.*, 2019). The overall protein representation $h_{pr}$ is obtained by applying the mean function on the representations of all amino acids:

| $h_{pr}=MEAN\left( h_{i}^{(2)} \vert\vert i\in all amino acid in protein \right)$ | (4) |
| --- | --- |

Where $h_{i}^{(2)}$ denotes the representation of the $i_{th}$ amino acid at the second (final) GIN layer, and $h_{pr}$ represents the protein graph representation. Then, this vector is passed through two fully connected layers, with a final sigmoid activation function used to predict the druggability score (Supplementary Fig. 5).

DNN-BERT and DNN-ESM. The DNN-BERT method is based on a deep neural network that utilizes BERT embeddings of protein sequences. BERT (Devlin *et al.*, 2019; Rao *et al.*, 2019) is a pre-trained language model that generates context-aware residue-level embeddings, with each embedding vector having a size of $768\times1$. By averaging the embeddings of all amino acids in a protein sequence, the BERT protein representation is obtained. This representation is then input into a deep neural network for classifying DT and non-DT proteins (Supplementary Fig. 6). The DNN-ESM method follows a similar structure but utilizes ESM-2 for generating sequence embeddings instead of BERT. However, because BERT has lower memory requirements compared to ESM-2, all proteins in the dataset can be processed by the DNN-BERT method without the need to remove tokens.

# References

Aleksander,S.A. *et al.* (2023) The Gene Ontology knowledgebase in 2023. *Genetics*, **224**, 1–14.

Ashburner,M. *et al.* (2000) Gene Ontology: tool for the unification of biology. *Nat. Genet.*, **25**, 25–29.

Bateman,A. *et al.* (2023) UniProt: the Universal Protein Knowledgebase in 2023. *Nucleic Acids Res.*, **51**, D523–D531.

Berman,H.M. (2000) The Protein Data Bank. *Nucleic Acids Res.*, **28**, 235–242.

Charles,R.Q. *et al.* (2017) PointNet: Deep Learning on Point Sets for 3D Classification and Segmentation. In, *2017 IEEE Conference on Computer Vision and Pattern Recognition (CVPR)*. IEEE, pp. 77–85.

Devlin,J. *et al.* (2019) BERT: Pre-training of Deep Bidirectional Transformers for Language Understanding. In, *Proceedings of the 2019 Conference of the North*. Association for Computational Linguistics, Stroudsburg, PA, USA, pp. 4171–4186.

Gligorijević,V. *et al.* (2021) Structure-based protein function prediction using graph convolutional networks. *Nat. Commun.*, **12**, 3168.

Huang,K. *et al.* (2020) SkipGNN: predicting molecular interactions with skip-graph networks. *Sci. Rep.*, **10**, 21092.

Knox,C. *et al.* (2024) DrugBank 6.0: the DrugBank Knowledgebase for 2024. *Nucleic Acids Res.*, **52**, D1265–D1275.

Meiler,J. *et al.* (2001) Generation and evaluation of dimension-reduced amino acid parameter representations by artificial neural networks. *J. Mol. Model.*, **7**, 360–369.

Rao,R. *et al.* (2019) Evaluating protein transfer learning with TAPE. *Adv. Neural Inf. Process. Syst.*, **32**, 1–13.

Wishart,D.S. (2006) DrugBank: a comprehensive resource for in silico drug discovery and exploration. *Nucleic Acids Res.*, **34**, D668–D672.

Xu,K. *et al.* (2019) How powerful are graph neural networks? *7th Int. Conf. Learn. Represent. ICLR 2019*, 1–17.


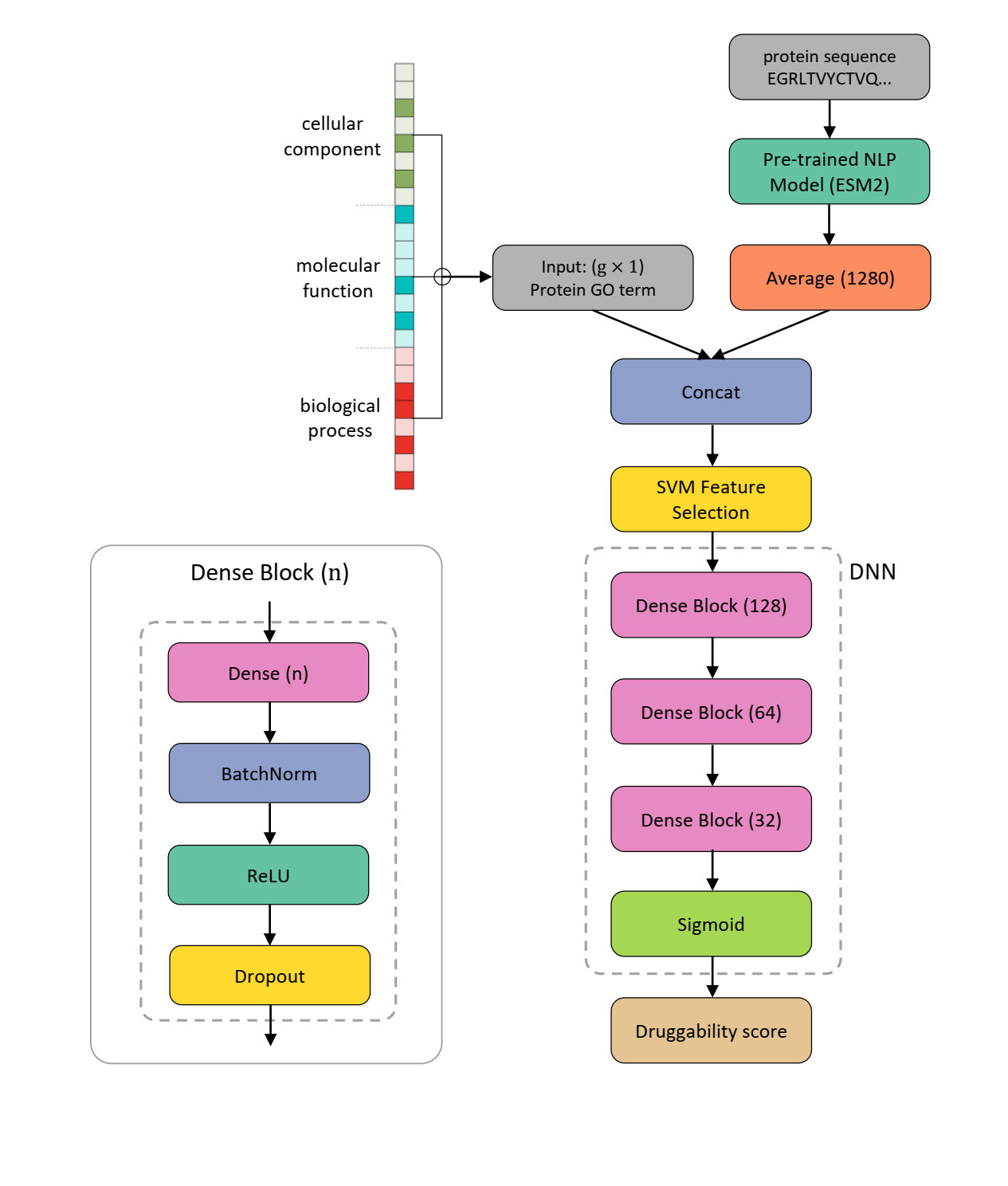


Supplementary Figure 1**- Schematic structure of DrugTar algorithm.**


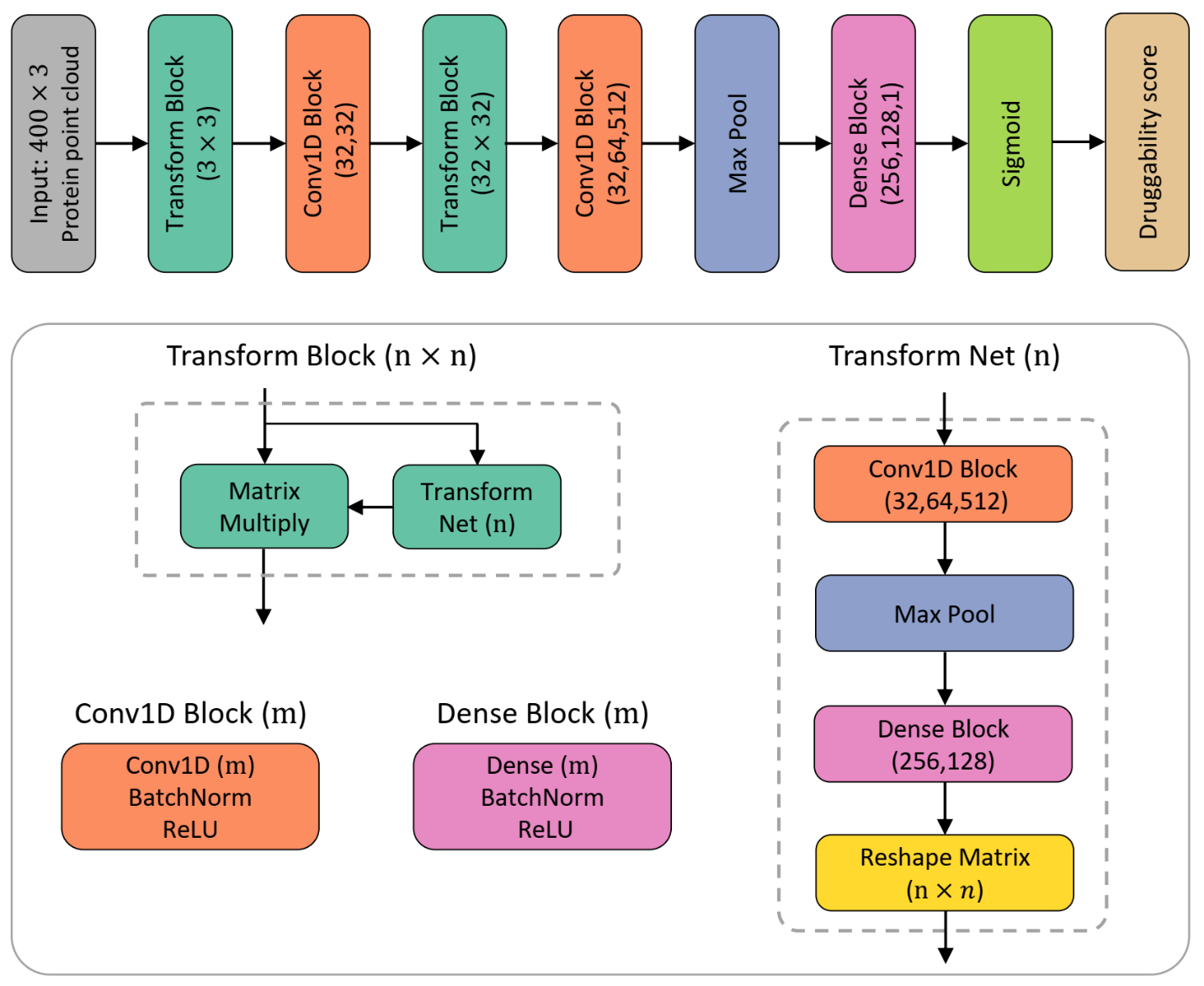


Supplementary Figure 2**-** **Overview of the PointNet-PC method framework.**


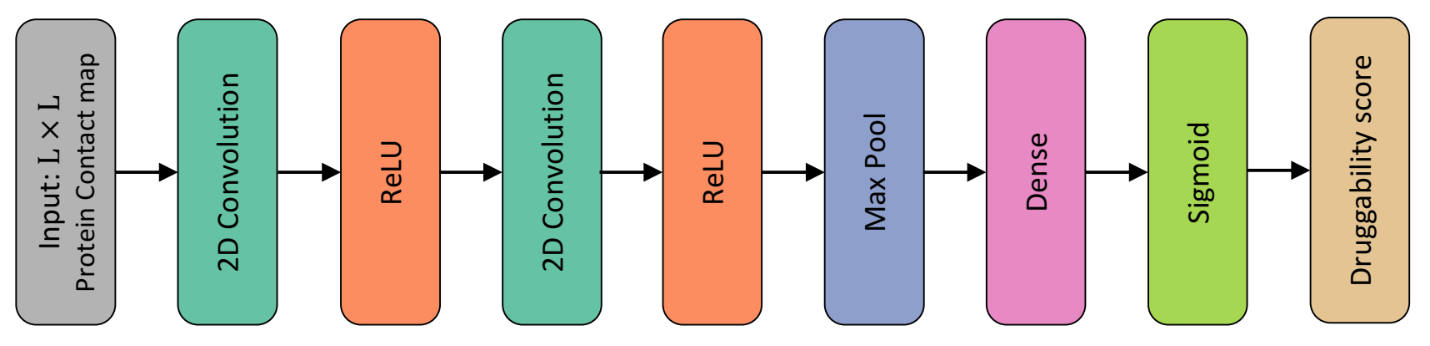


Supplementary Figure 3**-** **Overview of the CNN2D-CM method framework.**


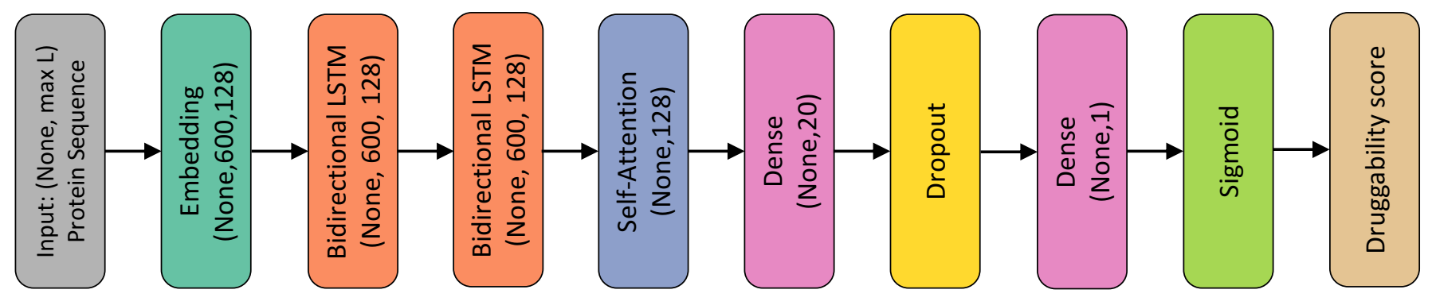


Supplementary Figure 4**- Overview of the RNN-DICT method framework.**


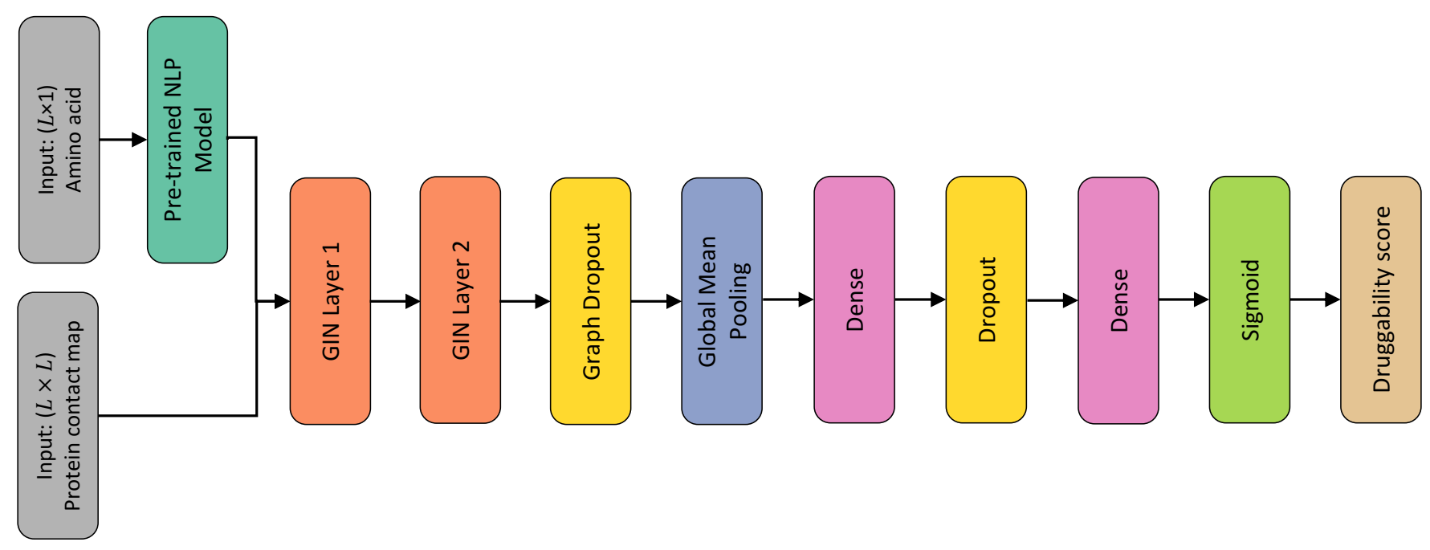


Supplementary Figure 5**-** **Overview of the GIN architecture in GNN-1H-PH and GNN-BERT method.**


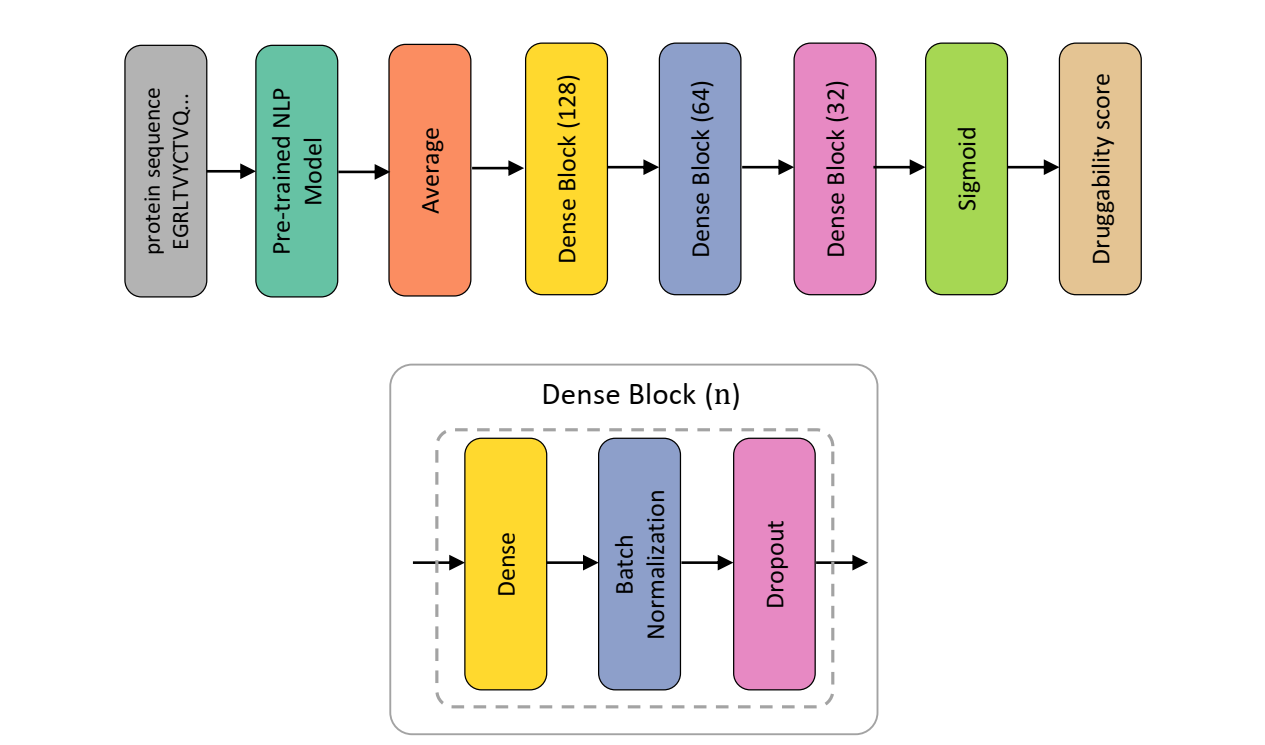


Supplementary Figure 6**- Overview of the DNN-BERT or DNN-ESM methods.**


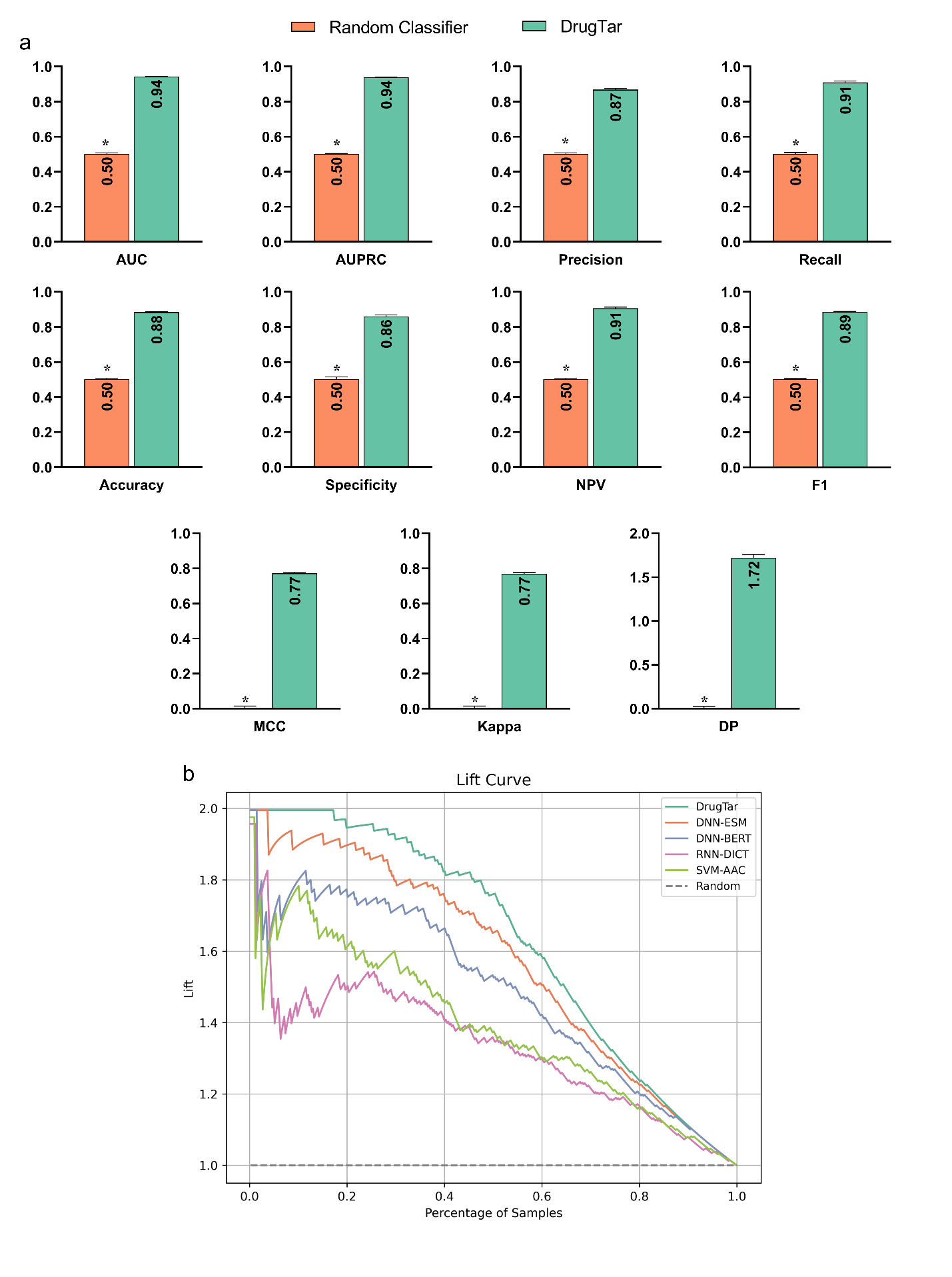


Supplementary Figure 7**-** **The performance of DrugTar was further assessed with the random classifier.** DrugTar was assessed against a random classifier across multiple indices, significantly outperforming it (P ≤ 0.05) (a). Lift curve analysis comparing DrugTar to sequence-based methods, showing superior performance. The dashed line represents random performance


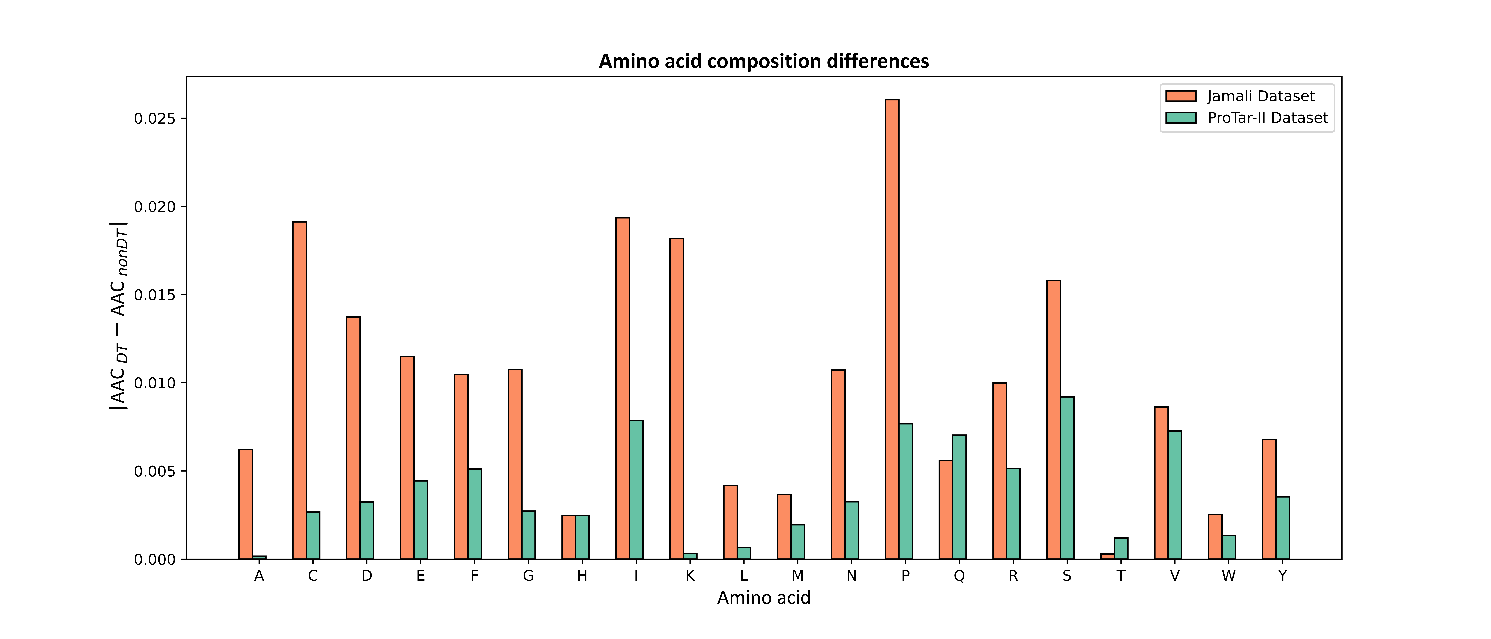


Supplementary Figure 8**-** **Differences** **in** **amino acid composition (AAC) between drug targets (DTs) and non-drug targets (non-DTs) in the Jamali and ProTar-II datasets.** The Jamali dataset exhibits significantly greater differences in AAC between DT and non-DT proteins compared to the ProTar-II dataset.
